# StandEnA: a customizable workflow for standardized annotation and generating a presence–absence matrix of proteins

**DOI:** 10.1093/bioadv/vbad069

**Published:** 2023-06-09

**Authors:** Fatma Chafra, Felipe Borim Correa, Faith Oni, Özlen Konu Karakayalı, Peter F Stadler, Ulisses Nunes da Rocha

**Affiliations:** Department of Environmental Microbiology, Helmholtz Centre for Environmental Research—UFZ, Leipzig 04318, Germany; Department of Molecular Biology and Genetics, Bilkent University, Ankara 06800, Turkey; Department of Environmental Microbiology, Helmholtz Centre for Environmental Research—UFZ, Leipzig 04318, Germany; Department of Computer Science and Interdisciplinary Center of Bioinformatics, University of Leipzig, Leipzig 04107, Germany; Department of Environmental Microbiology, Helmholtz Centre for Environmental Research—UFZ, Leipzig 04318, Germany; Department of Computer Science and Interdisciplinary Center of Bioinformatics, University of Leipzig, Leipzig 04107, Germany; Department of Molecular Biology and Genetics, Bilkent University, Ankara 06800, Turkey; Interdisciplinary Program in Neuroscience, Bilkent University, Ankara 06800, Turkey; UNAM-Institute of Materials Science and Nanotechnology, Bilkent University, Ankara 06800, Turkey; Department of Computer Science and Interdisciplinary Center of Bioinformatics, University of Leipzig, Leipzig 04107, Germany; Interdisciplinary Center for Bioinformatics, German Center for Integrative Biodiversity Research (iDiv) Halle-Jena-Leipzig, Competence Center for Scalable Data Services and Solutions, Leipzig Research Center for Civilization Diseases, Leipzig Research Center for Civilization Diseases (LIFE), University of Leipzig, Leipzig 04109, Germany; Max Planck Institute for Mathematics in the Sciences, Leipzig 04103, Germany; Institute for Theoretical Chemistry, University of Vienna, Vienna 1090, Austria; Facultad de Ciencias, Universidad National de Colombia, Sede Bogotá 111711, Colombia; Santa Fe Institute, Santa Fe, NM 87501, USA; Department of Environmental Microbiology, Helmholtz Centre for Environmental Research—UFZ, Leipzig 04318, Germany; Department of Computer Science and Interdisciplinary Center of Bioinformatics, University of Leipzig, Leipzig 04107, Germany

## Abstract

**Motivation:**

Several genome annotation tools standardize annotation outputs for comparability. During standardization, these tools do not allow user-friendly customization of annotation databases; limiting their flexibility and applicability in downstream analysis.

**Results:**

StandEnA is a user-friendly command-line tool for Linux that facilitates the generation of custom databases by retrieving protein sequences from multiple databases. Directed by a user-defined list of standard names, StandEnA retrieves synonyms to search for corresponding sequences in a set of public databases. Custom databases are used in prokaryotic genome annotation to generate standardized presence–absence matrices and reference files containing standard database identifiers. To showcase StandEnA, we applied it to six metagenome-assembled genomes to analyze three different pathways.

**Availability and implementation:**

StandEnA is an open-source software available at https://github.com/mdsufz/StandEnA.

**Supplementary information:**

Supplementary data are available at *Bioinformatics Advances* online.

## 1 Introduction

Protein annotation can be performed using different query databases; for example, the Universal Protein Resource Knowledgebase (UniProtKB) (Bairoch *et al.*, 2005; UniProt Consortium, 2017) and National Center for Biotechnology Information (NCBI) Entrez (NCBI ResourceCoordinators, 2013; Schuler *et al.*, 1996; Pruitt *et al.*, 2020). Most of the annotation in public repositories is user-dependent, leading to a lack of standardization of protein annotation across different repositories. The issue results in multiple redundant synonyms for the same protein. What is a significant hurdle when users need to compare annotations (Kalkatawi *et al.*, 2015; Klimke *et al.*, 2011). Hence, genome annotation pipelines strive to generate standardized outputs to include the metadata of standard database identifiers (Schwengers *et al.*, 2021).

Annotation tools, such as Bakta (Schwengers *et al.*, 2021) and MicrobeAnnotator (Ruiz-Perez *et al.*, 2021), attempt to standardize genome annotations in well-established tools such as Prokka (Seemann, 2014) and DFAST (Tanizawa *et al.*, 2018). While solving this problem, these tools trade user-friendly customizability for automation. Although existing tools provide some flexibility by allowing annotation using custom databases, users cannot use custom protein names to retrieve sequence files and generate custom databases within the annotation workflow. If users are interested in one particular pathway, compiling a custom database from various external databases and forming a reference file to match each enzyme synonym to its standard name is time-consuming and labor-intensive. Thus, users currently have limited ability to customize annotation databases to fit their individual needs.

We developed the Standardized Enzyme Annotation pipeline (StandEnA) to overcome nomenclature and customizability problems in pathway annotation. StandEnA is a user-friendly command-line tool for Linux. It annotates pathways selected by users on prokaryotic genomes by generating a user-defined custom database of protein sequences from available protein synonyms and standard names (Bairoch *et al.*, 2005; Haft *et al.*, 2003; Kanehisa *et al.*, 2017; Kanehisa and Goto, 2000; Kans, 2022; Kawashima *et al.*, 2003; Mistry *et al.*, 2021; O’Leary *et al.*, 2016; Pruitt *et al.*, 2005; Saraiva *et al.*, 2021; Sayers *et al.*, 2022; Schuler *et al.*, 1996; Seemann, 2014; Sonnhammer *et al.*, 1997; UniProt Consortium, 2021). To allow StandEnA’s annotations to be used in various downstream applications, genome annotation databases (NCBI Entrez and KEGG), sequence databases (NCBI RefSeq and UniProt), as well as protein family and domain databases (TIGRFAMs and Pfam) are used in this step (Supplementary Table S1) (Chen *et al.*, 2017). Thus, StandEnA appends custom database generation and genome annotation steps within the same workflow. The output is a presence-absence matrix of pathway enzymes and reference file identifiers from the Kyoto Encyclopedia of Genes and Genomes (KEGG) (Kanehisa, 1997; Kanehisa *et al.*, 2017) and Enzyme Commission (EC) (Enzyme nomenclature, 1993) from which users may customize the final nomenclature. To showcase the efficiency of our method, we tested StandEnA on six metagenome-assembled genomes (MAGs) using three different pathways.

## 2 Implementation

**Figure 1.**
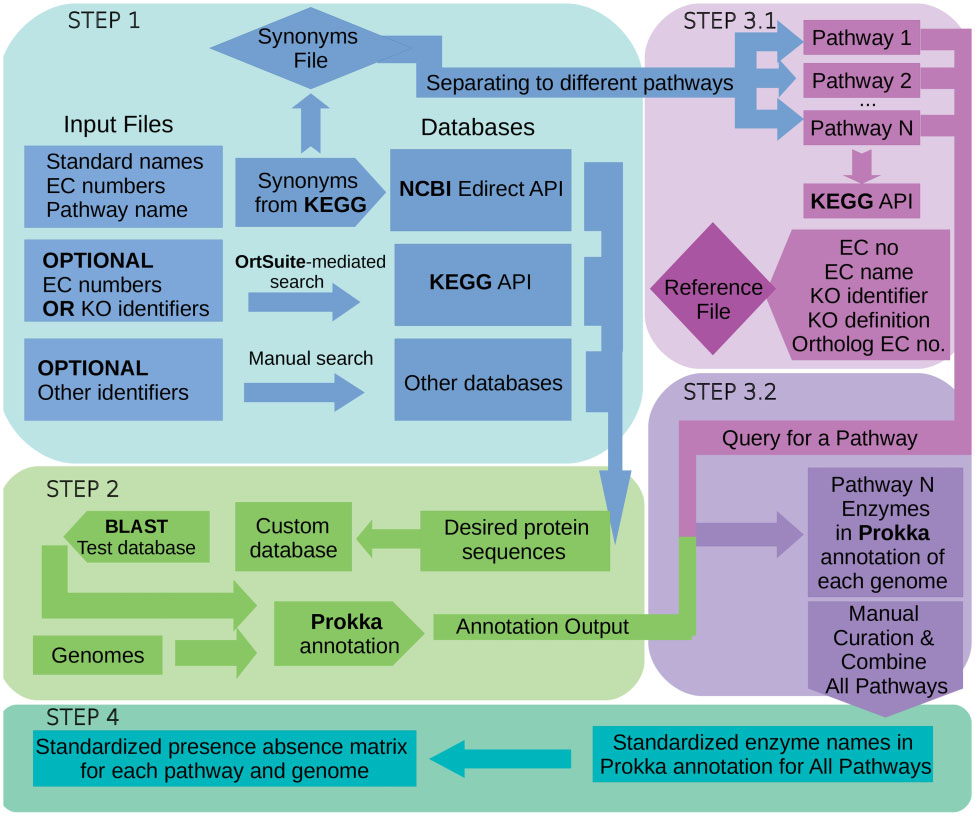
Workflow of StandEnA. Starting with enzyme identifiers for the pathways of interest, StandEnA has four steps, as follows: In Step 1, it compiles enzyme synonyms and identifiers for these pathways from various databases. After, Step 2 creates a custom database from these enzyme protein sequences and annotates genomes using this. Once Step 2 is completed, Step 3 creates a reference file with cross-database identifiers for each enzyme synonym used in the annotation and lists all of the enzymes of interest within the annotated genomes. Finally, Step 4 generates a standardized presence-absence matrix for each enzyme within the pathway of interest for these genomes

We implemented StandEnA using four custom scripts written in Python, Perl and Bash programming languages and grouped them in four main steps (Fig. 1) as Linux command-line scripts. System requirements depend on the genome number and size. We tested our pipeline using three genomes (NCBI accession numbers: CP021731.1, GCA_900092355.1 and NZ_VYSB01000001.1) for three pathways (Supplementary Files S1–S4 and Supplementary Table S2). On average, each annotated genome produces approximately 0.5–2 GB of data making disk space the most limiting resource. Additionally, *Escherichia coli* K12 genome (NC_000913.3) was annotated using StandEnA for the same three pathways and compared with annotations made using Prokka’s default database, the former revealing 3 times more standard enzyme annotations (Supplementary Files S5 and S6 and Supplementary Tables S3–S5). Furthermore, the process can take advantage of multicore parallelization and an alternative genome annotation step to analyze a large number of genomes simultaneously.

In Step 1, StandEnA creates a custom database using a user-provided input file containing standard enzyme names, their EC numbers, and KEGG pathway names (Kanehisa *et al.*, 2017). StandEnA then uses the KEGG database (Kanehisa *et al.*, 2017) to retrieve synonyms of these enzymes, which are used to download desired protein sequences from NCBI (NCBI Resource Coordinators, 2017). Optionally, proteins can be downloaded either directly from KEGG via an OrtSuite command (Saraiva *et al.*, 2021) using EC numbers or KEGG Orthology (KO) identifiers or manually from alternative databases. Users can monitor every input and output file directly, providing flexibility and preventing clashes with nomenclature conventions. In Step 2, genome annotation is performed by Prokka using the custom database with an option of incorporating Prokka’s default database (Haft *et al.*, 2003; Pruitt *et al.*, 2005; Seemann, 2014; Sonnhammer *et al.*, 1997).

Step 3 is divided into two parts. First, a reference file for each pathway is prepared. This file contains the KO identifier, EC number and standard name for each enzyme synonym used. After, the Prokka annotation output is searched for compiled enzyme synonym names within each pathway. Users can curate the synonyms from the Prokka annotation for further flexibility to remove any undesired protein name. Finally, Step 4 generates a standardized presence-absence matrix output for each pathway, including all of the genomes. Detailed information on input and output files is provided in Supplementary Figure S1.

## 3 Application

We analyzed our pipeline using six MAGs (accession numbers of these MAGs can be found in Supplementary Table S6) recovered from a benzene-degrading consortium (Eziuzor *et al.*, 2022) using MuDoGeR (da Rocha *et al.*, 2022). For this, we chose three different pathways (benzene degradation, catechol degradation and dissimilatory nitrate reduction pathways; Supplementary Files S2–S4). We fed the input file with standard enzyme names for each pathway (Supplementary Table S7) along with MAG file paths to the workflow. Without any manual curation step, StandEnA generated a preliminary custom database, annotated the genomes and outputted a presence-absence matrix containing results for all enzymes within three pathways (Supplementary File S7 and Supplementary Table S8, enzyme IDs Supplementary File S8). After the automated search, we suggest that users manually curate the database outputted by StandEnA via the StandEnA manual sequence download steps (refer to Fig. 1) to increase annotation comprehensiveness.

We demonstrated that manual search using the UniProt database could expand StandEnA’s initial custom database and fine-tune its synonyms list providing access to desired protein sequences that are unavailable in StandEnA’s default search (refer to Supplementary Table S1). Comparison between StandEnA annotations before and after manual curation (Supplementary Files S7–S9 and Supplementary Tables S8 and S9) for the six MAGs revealed that the default StandEnA search reproduced 46.4% of the annotation output after manual curation (Supplementary Tables S10 and S11). Additionally, 5.7% of the annotations were only found by the preliminary database, possibly due to changes in the synonym list after manual curation.

For six MAGs and three NCBI genomes, the entire pipeline was executed in under 8 h using a dual-core Intel Core i5 7th generation computer running Ubuntu 18.04.6 LTS on only 15 GB of free disk space. Compared to manual protein database construction, often reported to encompass a range of days to weeks (Blakeley-Ruiz and Kleiner, 2022), StandEnA generates significant time benefits while still generating a significant portion of the manual annotation results.

## 4 Conclusions

In this application note, we presented StandEnA, a customizable and standardized Linux command-line tool for annotating desired pathways in prokaryotic genomes via user-defined custom databases. StandEnA creates outputs containing a standardized presence–absence matrix of pathway enzymes and a reference file of standard database identifiers for each enzyme synonym used during annotation. The workflow conveniently creates a custom database containing desired protein sequence files from multiple databases. StandEnA provides customizability to genome annotations, as users can monitor all intermediate files and manually curate them when necessary. Manual curation improved the annotation of the analyzed pathways. Moreover, our tool performs database cross-references standardizing the outputs for simple presence–absence matrix comparisons across genomes, facilitating downstream utilization such as genomic/functional potential comparisons through metabolic pathway predictions and feature extraction for machine learning applications across different studies.

## Supplementary Material

vbad069_Supplementary_Data

## Data Availability

The data underlying this article are available as follows: 1. All metagenome-assembled genomes are available on European Nucleotide Archive (ENA) through the accession numbers GCA_946997315, GCA_946998175, GCA_946998845, GCA_946999225, GCA_946999665 and GCA_947000185. 2. All reference genomes are available on National Center for Biotechnology Information (NCBI) through the accession numbers CP021731.1, GCA_900092355.1 and NZ_VYSB01000001.1.
