# Supplementary material for: StandEnA: a customizable workflow for standardized annotation and generating a presence–absence matrix of proteins
Source: Bioinform Adv. 2023 Jun 9;3(1):vbad069. doi: 10.1093/bioadv/vbad069 (PMC10336186; doi:10.1093/bioadv/vbad069)
Supplement: vbad069_Supplementary_Data [file vbad069_supplementary_data.zip › Chafra_StandEnA_supplementary_table_1.docx]

**Supplementary Table 1.** Databases used for annotation by StandEnA’s default search

The databases used by StandEnA’s default search and the tools used to access them are detailed in the table. The database name, database reference, step of StandEnA that uses the database, tools used to access the database, tool reference, and the abbreviation of the tool and database is provided in each column.

| Database | Database Reference | Step of StandEnA that uses the database | Tool used to access database | Tool Reference | Abbreviation |
| --- | --- | --- | --- | --- | --- |
| National Center for Biotechnology Information (NCBI) Entrez | [[1,2]](https://www.zotero.org/google-docs/?fWKdFF) | Step 1 | National Center for Biotechnology Information Entrez Direct | [[3]](https://www.zotero.org/google-docs/?iGDZYO) | NCBI E-direct |
| Kyoto Encyclopedia of Genes and Genomes (KEGG) | [[4]](https://www.zotero.org/google-docs/?JEtTWy) | Step 1 | Kyoto Encyclopedia of Genes and Genomes Application Programming Interface | [[5]](https://www.zotero.org/google-docs/?UZjklV) | KEGG API |
| Kyoto Encyclopedia of Genes and Genomes (KEGG) | [[6]](https://www.zotero.org/google-docs/?Xty8Aq) | Step 1 | OrtSuite | [[7]](https://www.zotero.org/google-docs/?8EE4a1) | OrtSuite-mediated KEGG |
| Universal Protein Resource (UniProt) | [[8,9]](https://www.zotero.org/google-docs/?j2HxEo) | Step 2 | Prokaryotic Genome Annotation (Prokka) | [[10]](https://www.zotero.org/google-docs/?5Eyjky) | Prokka default database containing UniProt |
| National Center for Biotechnology Information Reference Sequences (RefSeq) | [[11,12]](https://www.zotero.org/google-docs/?T16dYG) | Step 2 | Prokaryotic Genome Annotation (Prokka) | [[10]](https://www.zotero.org/google-docs/?IdGXPh) | Prokka default database containing RefSeq |
| Protein Families Database (Pfam) | [[13,14]](https://www.zotero.org/google-docs/?hW1vt0) | Step 2 | Prokaryotic Genome Annotation (Prokka) | [[10]](https://www.zotero.org/google-docs/?SOXxbr) | Prokka default database containing Pfam |
| The Institute for Genomic Research Protein Families Database (TIGRFAMs) | [[15]](https://www.zotero.org/google-docs/?ilpd7D) | Step 2 | Prokaryotic Genome Annotation (Prokka) | [[10]](https://www.zotero.org/google-docs/?Umy0p7) | Prokka default database containing TIGRFAMs |

References

[1. Schuler GD, Epstein JA, Ohkawa H, Kans JA. [10] Entrez: Molecular biology database and retrieval system. Methods Enzymol [Internet]. Academic Press; 1996 [cited 2022 Oct 9]. p. 141–62. Available from: https://www.sciencedirect.com/science/article/pii/S0076687996660121](https://www.zotero.org/google-docs/?nIlU0H)

[2. Sayers EW, Bolton EE, Brister JR, Canese K, Chan J, Comeau DC, et al. Database resources of the national center for biotechnology information. Nucleic Acids Res. 2022;50:D20–6.](https://www.zotero.org/google-docs/?nIlU0H)

[3. Kans J. Entrez Direct: E-utilities on the Unix Command Line [Internet]. Entrez Program. Util. Help Internet. National Center for Biotechnology Information (US); 2022 [cited 2022 Oct 10]. Available from: https://www.ncbi.nlm.nih.gov/books/NBK179288/](https://www.zotero.org/google-docs/?nIlU0H)

[4. Kanehisa M, Goto S. KEGG: Kyoto Encyclopedia of Genes and Genomes. Nucleic Acids Res. 2000;28:27–30.](https://www.zotero.org/google-docs/?nIlU0H)

[5. Kawashima S, Katayama T, Sato Y, Kanehisa M. KEGG API: A Web Service Using SOAP/WSDL to Access the KEGG System. Genome Inform. 2003;14:673–4.](https://www.zotero.org/google-docs/?nIlU0H)

[6. Kanehisa M, Furumichi M, Tanabe M, Sato Y, Morishima K. KEGG: new perspectives on genomes, pathways, diseases and drugs. Nucleic Acids Res. Oxford Academic; 2017;45:D353–61.](https://www.zotero.org/google-docs/?nIlU0H)

[7. Saraiva JP, Bartholomäus A, Kallies R, Gomes M, Bicalho M, Kasmanas JC, et al. OrtSuite: from genomes to prediction of microbial interactions within targeted ecosystem processes. Life Sci Alliance [Internet]. Life Science Alliance; 2021 [cited 2022 Jun 21];4. Available from: https://www.life-science-alliance.org/content/4/12/e202101167](https://www.zotero.org/google-docs/?nIlU0H)

[8. Bairoch A, Apweiler R, Wu CH, Barker WC, Boeckmann B, Ferro S, et al. The Universal Protein Resource (UniProt). Nucleic Acids Res. 2005;33:D154–9.](https://www.zotero.org/google-docs/?nIlU0H)

[9. UniProt Consortium. UniProt: the universal protein knowledgebase in 2021. Nucleic Acids Res. 2021;49:D480–9.](https://www.zotero.org/google-docs/?nIlU0H)

[10. Seemann T. Prokka: rapid prokaryotic genome annotation. Bioinformatics. Oxford Academic; 2014;30:2068–9.](https://www.zotero.org/google-docs/?nIlU0H)

[11. Pruitt KD, Tatusova T, Maglott DR. NCBI Reference Sequence (RefSeq): a curated non-redundant sequence database of genomes, transcripts and proteins. Nucleic Acids Res. 2005;33:D501–4.](https://www.zotero.org/google-docs/?nIlU0H)

[12. O’Leary NA, Wright MW, Brister JR, Ciufo S, Haddad D, McVeigh R, et al. Reference sequence (RefSeq) database at NCBI: current status, taxonomic expansion, and functional annotation. Nucleic Acids Res. 2016;44:D733-745.](https://www.zotero.org/google-docs/?nIlU0H)

[13. Sonnhammer ELL, Eddy SR, Durbin R. Pfam: A comprehensive database of protein domain families based on seed alignments. Proteins Struct Funct Bioinforma. 1997;28:405–20.](https://www.zotero.org/google-docs/?nIlU0H)

[14. Mistry J, Chuguransky S, Williams L, Qureshi M, Salazar GA, Sonnhammer ELL, et al. Pfam: The protein families database in 2021. Nucleic Acids Res. 2021;49:D412–9.](https://www.zotero.org/google-docs/?nIlU0H)

[15. Haft DH, Selengut JD, White O. The TIGRFAMs database of protein families. Nucleic Acids Res. 2003;31:371–3.](https://www.zotero.org/google-docs/?nIlU0H)
