# Supplementary material for: StandEnA: a customizable workflow for standardized annotation and generating a presence–absence matrix of proteins
Source: Bioinform Adv. 2023 Jun 9;3(1):vbad069. doi: 10.1093/bioadv/vbad069 (PMC10336186; doi:10.1093/bioadv/vbad069)
Supplement: vbad069_Supplementary_Data [file vbad069_supplementary_data.zip › Chafra_StandEnA_supplementary_table_5_new.docx]

**Supplementary Table 5.** Comparison summary table between annotation outputs using automated StandEnA custom database (Supplementary Table 4) and Prokka default database (Supplementary Table 3). Summary table for the comparison indicating 4 categories and the number of standard enzyme annotations for each: Present when annotated with Prokka default database and automated StandEnA custom database, Absent when annotated with Prokka default database and automated StandEnA custom database, Present when annotated with automated StandEnA custom database but absent for Prokka default database annotation, Present when annotated with Prokka default database but absent for automated StandEnA custom database annotation. “Present when annotated with Prokka default database and automated StandEnA custom database” and “present when annotated with automated StandEnA custom database but absent for Prokka default database annotation” numbers were used to calculate the fold change in the number of standard annotations in the Implementation subheading.

| **Category Description** | **Number of standard enzyme annotations** |
| --- | --- |
| Present when annotated with Prokka default database and automated StandEnA custom database | 6 |
| Absent when annotated with Prokka default database and automated StandEnA custom database | 8 |
| Present when annotated with automated StandEnA custom database but absent for Prokka default database annotation | 18 |
| Present when annotated with Prokka default database but absent for automated StandEnA custom database annotation | 0 |
