# Supplementary material for: StandEnA: a customizable workflow for standardized annotation and generating a presence–absence matrix of proteins
Source: Bioinform Adv. 2023 Jun 9;3(1):vbad069. doi: 10.1093/bioadv/vbad069 (PMC10336186; doi:10.1093/bioadv/vbad069)
Supplement: vbad069_Supplementary_Data [file vbad069_supplementary_data.zip › Chafra_StandEnA_supplementary_table_6_new.docx]

**Supplementary Table 6.** European Nucleotide Archive (ENA) Metadata for the six metagenome-assembled genomes (MAGs) analyzed in this study. The sequences and the other metadata for the 6 MAGs analyzed in this study can be accessed through the ENA website using the information in this table. The column structure is as follows: assembly name of the genome, taxonomy (domain, phylum, class, order, family, genus, species), ENA assembly accession number, ENA study ID which the genomes have been submitted under, ENA sample ID, and the ENA contig accession number within the whole genome sequencing (WGS) sequence set of the sample. NA signifies not available.

| Assembly Name | Domain | Phylum | Class | Order | Family | Genus | Species | Assembly Accession Number | Study ID | Sample ID | Contig Accession |
| --- | --- | --- | --- | --- | --- | --- | --- | --- | --- | --- | --- |
| UMB088BNS | Bacteria | Planctomycetota | Brocadiae | Brocadiales | Brocadiaceae | Kuenenia | NA | GCA_946997315 | PRJEB41001 | ERS13477534 | CAMPVI010000001-CAMPVI010000357 |
| UMB007ABE | Bacteria | Proteobacteria | Alphaproteobacteria | Rhizobiales | Kaistiaceae | Bauldia | NA | GCA_946998175 | PRJEB41001 | ERS13477536 | CAMPYL010000001-CAMPYL010000184 |
| UMB132CIJ | Bacteria | Firmicutes B | Desulfitobacteriia | Desulfitobacteriales | Desulfitobacteriacea | UBA2264 | NA | GCA_946998845 | PRJEB41001 | ERS13477542 | CAMQBB010000001-CAMQBB010000182 |
| UMB155CQY | Bacteria | Firmicutes B | NA | NA | NA | NA | NA | GCA_946999225 | PRJEB41001 | ERS13477534 | CAMQCP010000001-CAMQCP010000049 |
| UMB071BFP | Bacteria | Proteobacteria | Gammaproteobacteria | Burkholderiales | Burkholderiaceae | Giesbergeria | Giesbergeria sp003096555 | GCA_946999665 | PRJEB41001 | ERS5289101 | CAMQDW010000001-CAMQDW010000049 |
| UMB102BVJ | Bacteria | Proteobacteria | Gammaproteobacteria | Burkholderiales | Burkholderiaceae | Giesbergeria | Giesbergeria sp003570885 | GCA_947000185 | PRJEB41001 | ERS13477545 | CAMQGD010000001-CAMQGD010000049 |
