# Supplementary material for: StandEnA: a customizable workflow for standardized annotation and generating a presence–absence matrix of proteins
Source: Bioinform Adv. 2023 Jun 9;3(1):vbad069. doi: 10.1093/bioadv/vbad069 (PMC10336186; doi:10.1093/bioadv/vbad069)
Supplement: vbad069_Supplementary_Data [file vbad069_supplementary_data.zip › Chafra_StandEnA_supplementary_table_7_new.docx]

**Supplementary Table 7.** KEGG database identifiers for the three pathways tested in this study. The Kyoto Encyclopedia of Genes and Genomes (KEGG) database identifiers for the three pathways tested in this study are provided in this table. The column structure is as follows: KEGG pathway description/name, enzyme commission (EC) number for each enzyme, KEGG Orthology (KO) identifier also called the K number for each enzyme, KEGG module number for each enzyme, and standard protein name within the KEGG database for each enzyme.

| **Pathway Description** | **EC number** | **KO identifier (K number)** | **Module number** | **Standard protein name** |
| --- | --- | --- | --- | --- |
| Aerobic degradation without activation by coenzyme A | 1.3.1.25 | K05783 | M00551 | 1,6-dihydroxycyclohexa-2,4-diene-1-carboxylate dehydrogenase |
|  | 1.14.12.10 | K05549 |  | benzoate 1,2-dioxygenase, alpha subunit |
|  | 1.14.12.10 | K05550 |  | benzoate 1,2-dioxygenase, beta subunit |
|  | 1.14.12.10 | K05784 |  | benzoate 1,2-dioxygenase, reductase component |
|  | 1.14.12.10 | NA |  | benzoate 1,2-dioxygenase (ambiguous) |
| Dissimilatory nitrate reduction to ammonium (nitrate reduction to nitrite) | 3.6.3.- | K15576 | M00615 | nitrate/nitrite transport system substrate-binding protein (nrtA) |
|  | 3.6.3.- | K15577 |  | nitrate/nitrite transport system permease protein (nrtB) |
|  | 3.6.3.- | K15578 |  | nitrate/nitrite transport system ATP-binding protein (nrtC) |
|  | 3.6.3.- | K15579 |  | nitrate/nitrite transport system ATP-binding protein (nrtD) |
|  | 3.6.3.- | K02575 |  | MFS transporter, NNP family, nitrate/nitrite transporter (NRT) |
|  | 1.7.1.2 | K10534 | M00531 | nitrate reductase (NAD(P)H) |
|  | 1.7.5.1 | K00370 | M00530 | nitrate reductase, alpha subunit (quinone) |
|  | 1.7.5.1 | K00371 |  | nitrate reductase, beta subunit (quinone) |
|  | 1.7.5.1 | K00374 |  | nitrate reductase, gamma subunit (quinone) |
|  | 1.7.5.1 | NA |  | nitrate reductase (quinone) (ambiguous) |
|  | 1.7.7.2 | K00367 | M00531 | ferredoxin-nitrate reductase |
|  | NA | K02567 |  | nitrate reductase (cytochrome) |
|  | 1.7.99.- | K02568 | M00530 | nitrate reductase (cytochrome), electron transfer subunit |
|  | 1.7.99.- | NA | NA | periplasmic nitrate reductase (other) |
| Aerobic degradation of catechol - ortho cleavage pathway | 1.13.11.1 | K03381 | M00568 | catechol 1,2-dioxygenase |
|  | 5.5.1.1 | K01856 |  | muconate cycloisomerase |
|  | 5.3.3.4 | K03464 |  | muconolactone isomerase |
|  | 3.1.1.24 | K01055, K14727 |  | 3-oxoadipate enol-lactonase |
|  | 2.8.3.6 | K01031 | NA | 3-oxoadipate CoA-transferase, alpha subunit |
|  | 2.8.3.6 | K01032 | NA | 3-oxoadipate CoA-transferase, beta subunit |
|  | 2.3.1.174 | K02615, K07823 | M00878 | 3-oxoadipyl-CoA thiolase |
