# Supplementary material for: StandEnA: a customizable workflow for standardized annotation and generating a presence–absence matrix of proteins
Source: Bioinform Adv. 2023 Jun 9;3(1):vbad069. doi: 10.1093/bioadv/vbad069 (PMC10336186; doi:10.1093/bioadv/vbad069)
Supplement: vbad069_Supplementary_Data [file vbad069_supplementary_data.zip › Chafra_StandEnA_Supplementary_table_10_new.docx]

**Supplementary Table 10**. Comparison table between annotation outputs using manually created custom database (Supplementary Table 9) and automated StandEnA database (Supplementary Table 8). The comparison table for the results generated after manual database curation, as described in the manuscript, for the same three pathways (refer to Supplementary Table 7) on the same 6 MAGs and the automated StandEnA output with the labels: SP for the same before and after manual curation (present); SA for the same before and after manual curation (absent); DM for different before and after manual curation (present after manual curation); and DS for different before and after manual curation (present before manual curation). No shading indicates the annotations with matching results before and after manual curation. Light gray shading is used for results where the enzyme is present after manual curation but not before, whereas dark gray shading is used for results where the enzyme is present before manual curation but not after. The column structure is as follows: standard name of the enzyme, protein ID for the enzyme used in this study (refer to Supplementary File 8), species information in the form of unique European Nucleotide Archive (ENA) assembly name (refer to Supplementary Table 6).

| Standard Name | Protein ID in this study | Protein ID in [REF] | Specie | | | | | |
| --- | --- | --- | --- | --- | --- | --- | --- | --- |
|  |  |  | UMB071 | UMB088 | UMB155 | UMB007 | UMB132 | UMB102 |
| catechol 1,2-dioxygenase | CP1001 | CP1021 | SA | DM | SA | DM | SA | DM |
| muconate cycloisomerase | CP1002 | CP1022 | DM | DM | SA | SP | DM | DM |
| muconolactone isomerase | CP1003 | CP1023 | SA | SA | SA | DM | DM | SA |
| 3-oxoadipate enol-lactonase | CP1004 | CP1024 | SP | DM | DM | SP | SA | DM |
| 3-oxoadipyl-coa thiolase | CP1005 | CP1027 | DM | SA | SA | SA | SA | DM |
| 3-oxoadipate coa-transferase, alpha subunit | CP1006 | CP1025 | DM | SA | SA | DM | DM | DM |
| 3-oxoadipate coa-transferase, beta subunit | CP1007 | CP1026 | SA | SA | SA | SP | DS | SA |
| 1,6-dihydroxycyclohexa-2,4-diene-1-carboxylate dehydrogenase | CP1008 | CP0020 | DM | DM | SA | DM | SA | SA |
| benzoate 1,2-dioxygenase, alpha subunit | CP1009 | CP0021 | SA | SA | SA | SA | SA | SA |
| benzoate 1,2-dioxygenase, beta subunit | CP1010 | CP0022 | SA | SA | SA | SA | SA | SA |
| benzoate 1,2-dioxygenase, reductase component | CP1011 | CP0023 | DM | SA | SA | DM | SA | DM |
| benzoate 1,2-dioxygenase (ambiguous) | CP1012 | CP0024 | SA | SA | SA | SA | SA | SA |
| nitrate reductase, alpha subunit (quinone) | CP1013 | CP0069 | DM | DM | DM | DM | DM | DM |
| nitrate reductase, beta subunit (quinone) | CP1014 | CP0070 | DM | DM | SA | DM | DM | DM |
| nitrate reductase, gamma subunit (quinone) | CP1015 | CP0071 | DM | SA | SA | DM | DM | DM |
| nitrate reductase quinone (ambiguous) | CP1016 | CP0072 | DM | DM | DM | DM | DM | DM |
| nitrate reductase (cytochrome) | CP1017 | CP0074 | DS | SA | DM | DS | SA | DS |
| nitrate reductase (cytochrome), electron transfer subunit | CP1018 | CP0075 | SA | SA | SA | SA | SA | SA |
| nitrate reductase (NAD(P)H) | CP1019 | CP0068 | DM | SA | SA | SA | SA | SA |
| ferrodoxin-nitrate reductase | CP1020 | CP0073 | SA | DM | DS | SA | DS | SA |
| nitrate/nitrite transport system substrate-binding protein (nrta) | CP1021 | CP0063 | SA | SA | SA | SA | SA | DM |
| nitrate/nitrite transport system permease protein (nrtb) | CP1022 | CP0064 | DM | SA | DM | DM | DM | DM |
| nitrate/nitrite transport system atp-binding protein (nrtc) | CP1023 | CP0065 | DM | DM | DM | DM | DM | DM |
| nitrate/nitrite transport system atp-binding protein (nrtd) | CP1024 | CP0066 | DM | DM | DM | DM | DM | DM |
| MFS transporter, NNP family, nitrate/nitrite transporter (NRT) | CP1025 | CP0067 | SA | SA | SA | SA | DM | SA |
| periplasmic nitrate reductase other | CP1026 | CP0076 | DM | DM | SA | DM | DM | SA |
| nitrite reductase (cytochrome c-552) | CP1027 | CP0083 | SA | DM | SP | SA | SP | SA |
| cytochrome c nitrite reductase small subunit | CP1028 | CP0084 | DM | DM | DM | SA | DM | SA |
| nitrite reductase (NADH), small subunit | CP1029 | CP0085 | DM | SA | SA | DM | DM | DM |
| nitrite reductase (NADH), large subunit | CP1030 | CP0086 | DM | DM | DM | DM | DM | DM |
| nitrite reductase (NAD(P)H) | CP1031 | CP0087 | DS | DS | SA | DS | DS | DS |
| ferredoxin-nitrite reductase | CP1032 | CP0088 | DM | DM | DM | DM | DM | DM |
