# Supplementary material for: StandEnA: a customizable workflow for standardized annotation and generating a presence–absence matrix of proteins
Source: Bioinform Adv. 2023 Jun 9;3(1):vbad069. doi: 10.1093/bioadv/vbad069 (PMC10336186; doi:10.1093/bioadv/vbad069)
Supplement: vbad069_Supplementary_Data [file vbad069_supplementary_data.zip › Chafra_StandEnA_supplementary_table_11_new.docx]

**Supplementary Table 11.** Comparison summary table between annotation outputs using manually created custom database (Supplementary Table 9) and automated StandEnA database (Supplementary Table 8).

Summary table for the comparison in Supplementary Table 10 containing the description of the category, its abbreviation, and the number of instances, totals, and percentages within the data for the four different categories in Supplementary Table 10. SP for the same before and after manual curation (present); SA for the same before and after manual curation (absent); DM for different before and after manual curation (present after manual curation); and DS for different before and after manual curation (present before manual curation).

| **Category Description** | **Abbreviation** | **Totals** | **Percentage** |
| --- | --- | --- | --- |
| Same in manual and automated StandEnA (present) | SP | 6 | 3.135 |
| Same in manual and automated StandEnA (absent) | SA | 83 | 43.23 |
| Different in manual and automated StandEnA (present in manual) | DM | 92 | 47.92 |
| Different in manual and automated StandEnA (present in automated) | DS | 11 | 5.73 |
