# Supplementary material for: StandEnA: a customizable workflow for standardized annotation and generating a presence–absence matrix of proteins
Source: Bioinform Adv. 2023 Jun 9;3(1):vbad069. doi: 10.1093/bioadv/vbad069 (PMC10336186; doi:10.1093/bioadv/vbad069)
Supplement: vbad069_Supplementary_Data [file vbad069_supplementary_data.zip › Chafra_StandEnA_supplementary_figure_1.pdf]

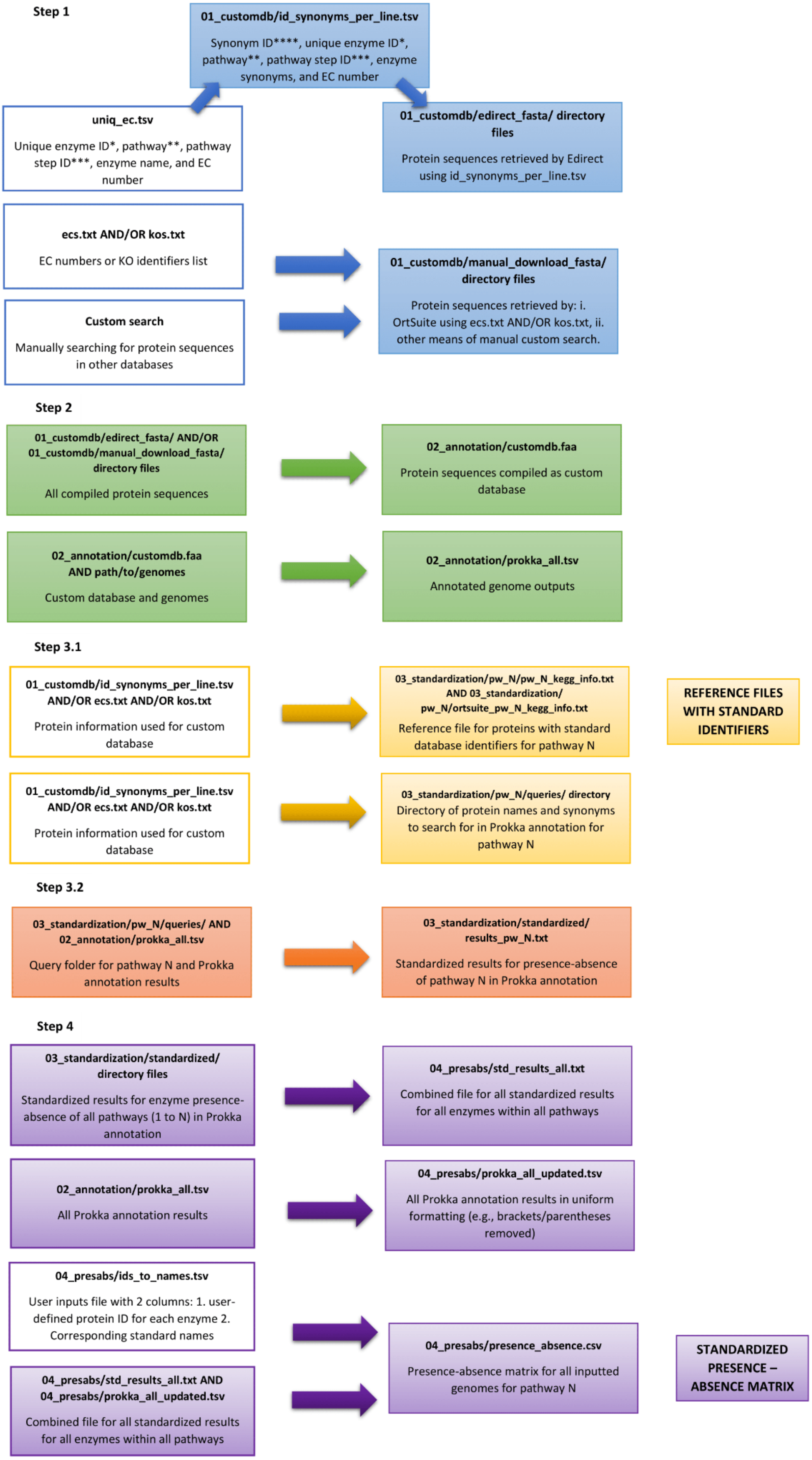
Step 1
01_customdb/id_synonyms_per_line.tsv
Synonym ID****, unique enzyme ID*, pathway**, pathway step ID***, enzyme synonyms, and EC number
uniq_ec.tsv
Unique enzyme ID*, pathway**, pathway step ID***, enzyme name, and EC number
01_customdb/edirect_fasta/ directory files
Protein sequences retrieved by Edirect using id_synonyms_per_line.tsv
ecs.txt AND/OR kos.txt
EC numbers or KO identifiers list
Custom search
Manually searching for protein sequences in other databases
01_customdb/manual_download_fasta/ directory files
Protein sequences retrieved by: i. OrtSuite using ecs.txt AND/OR kos.txt, ii. other means of manual custom search.
Step 2
01_customdb/edirect_fasta/ AND/OR 01_customdb/manual_download_fasta/ directory files
All compiled protein sequences
02_annotation/customdb.faa
Protein sequences compiled as custom database
02_annotation/customdb.faa AND path/to/genomes
Custom database and genomes
02_annotation/prokka_all.tsv
Annotated genome outputs
Step 3.1
01_customdb/id_synonyms_per_line.tsv AND/OR ecs.txt AND/OR kos.txt
Protein information used for custom database
03_standardization/pw_N/pw_N_kegg_info.txt AND 03_standardization/ pw_N/ortsuite_pw_N_kegg_info.txt
Reference file for proteins with standard database identifiers for pathway N
REFERENCE FILES WITH STANDARD IDENTIFIERS
01_customdb/id_synonyms_per_line.tsv AND/OR ecs.txt AND/OR kos.txt
Protein information used for custom database
03_standardization/pw_N/queries/ directory
Directory of protein names and synonyms to search for in Prokka annotation for pathway N
Step 3.2
03_standardization/pw_N/queries/ AND 02_annotation/prokka_all.tsv
Query folder for pathway N and Prokka annotation results
03_standardization/standardized/ results_pw_N.txt
Standardized results for presence-absence of pathway N in Prokka annotation
Step 4
03_standardization/standardized/ directory files
Standardized results for enzyme presence-absence of all pathways (1 to N) in Prokka annotation
04_presabs/std_results_all.txt
Combined file for all standardized results for all enzymes within all pathways
02_annotation/prokka_all.tsv
All Prokka annotation results
04_presabs/prokka_all_updated.tsv
All Prokka annotation results in uniform formatting (e.g., brackets/parentheses removed)
04_presabs/ids_to_names.tsv
User inputs file with 2 columns: 1. user-defined protein ID for each enzyme 2. Corresponding standard names
04_presabs/std_results_all.txt AND 04_presabs/prokka_all_updated.tsv
Combined file for all standardized results for all enzymes within all pathways
04_presabs/presence_absence.csv
Presence-absence matrix for all inputted genomes for pathway N
STANDARDIZED PRESENCE – ABSENCE MATRIX

Supplementary Figure 1. StandEnA Input and Output Files for each step

Figure detailing the input and output file names and contents for each step of StandEnA. The column structure of the figure is as follows: (1) input file name and description; (2) output file name and description; (3) description of the output's significance for the pipeline. Color-filled boxes indicate files generated by StandEnA, whereas the white background boxes indicate the files manually inputted by the user. Each input-output file pair is ordered according to its sequence of appearance in the pipeline, and it can be seen under the corresponding main step number in line with Figure 1.
